# Are Blood Pressure and Cardiovascular Stress Greater in Isometric or in Dynamic Resistance Exercise?

**DOI:** 10.3390/sports8040041

**Published:** 2020-03-28

**Authors:** Anastasios Kounoupis, Stavros Papadopoulos, Nikiforos Galanis, Konstantina Dipla, Andreas Zafeiridis

**Affiliations:** 1 Laboratory of Exercise Physiology and Biochemistry, Department of Physical Education and Sports Sciences at Serres, Aristotle University of Thessaloniki, Ippokratous 22, Ag. Ioannis, 62110 Serres, Greece; anastkounoupis@hotmail.com (A.K.); papas976@hotmail.com (S.P.); kdipla@phed-sr.auth.gr (K.D.); 2 School of Medicine, Aristotle University of Thessaloniki, 54124 Thessaloniki, Greece; kyros@auth.gr

**Keywords:** isometric, static, dynamic, resistance, exercise, blood pressure, heartrate, cardiovascular, metaboreflex, contraction

## Abstract

Medical and sports medicine associations are reluctant to endorse isometric exercise to the same extent as dynamic resistance exercise (RE). The major concern is the fear of greater increases in blood pressure (BP) that might be associated with isometric exercise. This review comprehensively presents all human studies that directly compared the magnitude of hemodynamic responses between isometric and dynamic RE. We also discuss possible mechanisms controlling BP-response and cardiovascular adjustments during both types of RE. The most prominent finding was that isometric and dynamic RE using small-muscle mass evoke equal increases in BP; however, the circulatory adjustments contributing to this response are different in dynamic and isometric RE. In contrast, studies using large-muscle mass report inconsistent results for the magnitude of BP-response between the two types of RE. Thus, when the same muscles and workloads are used, the increase in BP during isometric and dynamic RE is more comparable to what is commonly believed. However, it should be noted that only a few studies equalized the workload in two types of RE, most used small sample sizes, and all studies employed healthy participants. More studies are needed to compare the cardiovascular risks associated with isometric and dynamic RE, especially in individuals with chronic disease.

## 1. Introduction

Resistance exercise (RE) has been considered an integral component of exercise training programs for the promotion of health [1,2]. In recent years, RE has been widely used in clinical settings for improving muscle performance and functional capacity, for prevention and treatment of chronic diseases as an adjunct to aerobic exercise as well as in rehabilitation of musculoskeletal injuries [2,3,4,5,6,7]. RE training causes central and peripheral adaptations to the human body, such as structural and morphological changes in the heart [1,3,8,9], improvements in vascular endothelial function [3,10], and reductions in resting blood pressure [3,4,6,10]. RE training has also been associated with favorable changes in body composition and muscle profile, such as increases in muscle mass and strength and improvements in glucose and fat metabolism, as well as in insulin sensitivity [1,2,3,10,11].

RE is performed using either dynamic or isometric (static) contractions. The two types of muscular contractions are characterized by different mechanical properties. Resistance isometric (static) contraction is manifested by an increase in muscle tension and force generation with no significant alterations in the muscle’s belly length and no limb movement, while dynamic (isotonic/isokinetic) contraction is characterized by the stretch-shortening cycle, where force is produced by varying the muscle length and causing the movement of the limb [12]. Sustained isometric contraction generates constant intramuscular pressure to the vasculature. This continuously impedes or even occludes blood flow, limits O_2_ delivery and oxidative metabolism, increases peripheral vascular resistance, and imposes significant pressure load on the circulatory system [3,13]. In contrast, during dynamic RE, blood flow is restricted during the contraction phase but increased during the relaxation phase, resulting in greater O_2_ delivery and oxidative metabolism [3,14,15]. The different blood flow patterns between dynamic and isometric contractions, as well as differences in oxygen consumption (increased in dynamic) and peripheral resistance (increased in isometric), may alter the magnitude of blood pressure (BP) and other cardiovascular responses during the two types of RE. Indeed, the higher intramuscular pressure and mechanical compression of the vascular compartment within the muscles during RE has been suggested as one of the main contributors to the greater exercise BP response [16].

Isometric compared to dynamic RE has been shown to confer comparable benefits in terms of muscle hypertrophy [17] and appears superior for angle-specific strength gains. Furthermore, isometric exercise training seems to elicit at least similar (if not greater) BP-lowering effects than dynamic resistance training [4,18,19] and could be of benefit to individuals with mobility issues to increase their muscle mass and strength. Despite this, medical and sports medicine associations as well as health practitioners are still reluctant to endorse isometric exercise to the same degree as dynamic resistance training. In fact, only recently the American Heart Association and the Exercise and Sport Science Australia Association have included isometric exercise in their guidelines as a complementary prevention and therapeutic strategy for hypertension [20,21,22]. The concerns associated with isometric exercise are possibly related to the fear of excessive cardiovascular responses, and particularly exaggerated increases in BP, which may represent a risk for adverse cerebrovascular and cardiac events [6,23,24,25,26,27,28].

The general perception that isometric produces greater BP response compared to dynamic exercise is mainly based on studies comparing isometric with moderate intensity dynamic aerobic exercise. To the best of the authors’ knowledge, there is no review summarizing studies that compared the hemodynamic response between isometric and dynamic RE. For this reason, the aim of this review was to present and discuss those human studies that directly compared the magnitude of BP and other cardiovascular responses during these two types of RE using the same muscle mass. The answer to this question is of considerable clinical importance given the increasing use of isometric exercise in healthy individuals and in individuals with chronic diseases, including those with heart disease and hypertension.

## 2. Methods

**Figure 1:**
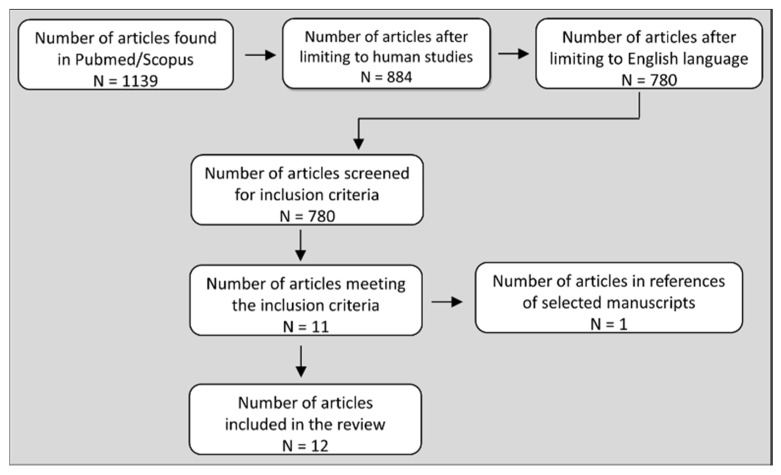
Flowchart showing the selection process of the articles presented in this review.

A review of the existing literature was conducted using the PubMed and Scopus databases to identify studies that directly (within the same design) compared changes in blood pressure and other hemodynamic parameters between isometric (static) and dynamic (isokinetic or isotonic) resistance exercises. These two search engines were selected as they are optimal tools in life sciences, physiology, and biomedicine. To search the database for relevant articles, we used the combinations of terms “isometric”, “static”, “resistance”, “isokinetic”, “isotonic”, “dynamic” with either “exercise” or “contraction” and with each of the following terms “blood pressure”, “cardiac output”, heartrate”, “stroke volume”, “peripheral resistance”, “systemic vascular resistance”, “blood flow”, and “vascular conductance”. Boolean operators “AND” and “OR” were used as conjunctions in the search. The search and the review of the articles were performed independently by 3 authors (A.K., K.D., and N.G.) in November of 2019 and updated in March of 2020. The title and abstract of each article were screened for suitability and the full-text articles were retrieved to determine the inclusion or the exclusion in the review. Inclusion criteria were: (i) original, English language research articles, (ii) human studies, (ii) both isometric and dynamic resistance (isokinetic or isotonic) exercise protocols were performed within the same experimental design for direct comparison, (iii) the same muscle group was exercised in the two types of RE protocols, (iv) BP was measured during RE protocols, (v) statistics were used to directly compare the BP responses between isometric and dynamic RE protocols. The exclusion criteria were: (i) the above inclusion criteria were not met and (ii) the conclusions made in the articles were uncertain (observed in one study). The references of the selected manuscripts were screened to identify additional articles that met the inclusion criteria. To our surprise, only 12 investigations in humans and one in animals (cat) [29] directly compared blood pressure and other hemodynamic responses between isometric and dynamic RE using the same muscle mass in humans. Two studies in humans were not included in the review because in one, the conclusions for the effects of isometric and dynamic RE on blood pressure were uncertain [30] and in another, statistical comparison between static and dynamic RE was not performed [31]. Figure 1 describes the process of selecting the articles that are presented in this review. 

## 3. Studies that Compared BP and Hemodynamic Responses between Isometric and Dynamic RE

This review includes only those studies that directly compared blood pressure and other hemodynamic responses between isometric and dynamic RE using the same muscle mass in humans. The study design characteristics and the results of manuscripts comparing BP as well as other hemodynamic responses between isometric and dynamic RE for small muscle and intermediate-large muscle mass protocols are presented in Table 1 and Table 2, respectively.

One of the first studies to directly compare dynamic and isometric (static) resistance exercise was performed by Lewis et al. [32]. In that study, the cardiovascular responses between static (25% of maximum voluntary contraction, MVC, 370 s) and dynamic (11 kg, 20–40 repetitions per min, 359 s) handgrip exercise performed to fatigue were compared in six healthy men. The authors concluded that isometric and dynamic handgrip exercise when performed to common local fatigue, produce equal increases in systolic BP (+32 versus +39 mmHg, respectively), diastolic BP (+24 versus +26 mmHg, respectively), mean BP (+26 versus +30 mmHg, respectively), and heart rate (+9 versus +24 beats/min, respectively), with no differences within and between RE protocols in stroke volume. Also, no differences were found in oxygen consumption and plasma noradrenaline levels between the two modes of contraction, despite their two-fold greater increase in dynamic than in static handgrip exercise. Cardiac output increased only in dynamic RE, whereas total peripheral resistance increased only in isometric RE, suggesting the involvement of different mechanisms controlling the BP response in two modes of RE using small muscle groups. In the same study, autonomic blockade abolished the heart rate response during static and dynamic RE, but the BP response was only slightly attenuated after combined β-adrenergic and parasympathetic blockade for both modes of contraction. This finding supports the involvement of muscle neural reflexes in the control of BP during both types of RE. In line, Louhevaara et al. [33] did not observe differences in BP and heart rate responses as well as in oxygen consumption between dynamic and isometric handgrip exercises in 21 healthy men. The results of the Louhevaara et al. [33] study, as well as in Lewis et al.’s study, are limited by the fact that the exercise intensity and the duration of dynamic and isometric handgrips were not matched. Specifically, the dynamic, compared to isometric, handgrip exercise was performed at significantly greater intensity (57% versus 46% MVC, respectively) and for longer duration (170 versus 99 s, respectively). In fact, Louhevaara et al., suggested that the approximately double exercise duration in the dynamic than in the isometric protocol was possibly the main contributing factor for the comparable BP and heart rate responses in two modes of contraction. In support to Lewis et al. [32], the researchers of the above study [33] concluded that when RE is performed using small muscle mass groups, the type of muscle contraction (dynamic versus isometric) is not the major determinant of the cardiorespiratory responses.

Two years later, Lewis and colleagues [34] attempted to characterize the role of muscle mass as a cofactor that may differentiate the hemodynamic response between the two modes of contraction. To answer this question, the participants performed isometric and dynamic RE protocols using small and large muscle groups (handgrip and two-legged knee extension) with both types of contraction performed to exhaustion. The authors hypothesized that the exercising muscle mass is the main contributor to the BP response, but the mode of contraction would influence the mechanisms of circulatory adjustments contributing to the BP response. Of note, the exercise time between dynamic RE and static RE was not matched. Their results showed that the magnitude of heart rate, BP, cardiac output, total peripheral resistance, and energy expenditure responses were greater during exercise with larger muscle mass, but the magnitude of BP response was not affected by the contraction mode. In line with their previous findings, the authors concluded that the similar BP responses during static and dynamic RE protocols, irrespective of muscle mass, were attributed to different circulatory adjustments. That is, due to an unchanged peripheral resistance and a rise in cardiac output during static RE and a drop in peripheral resistance with proportionately larger increases in cardiac output in the dynamic RE protocol. Considering the greater responses in stroke volume, cardiac output, and total peripheral resistance in dynamic RE than in static RE, Lewis et al. [34] speculated that the similar changes in heart rate and mean BP may be due to the modulating effect of two modes of contraction on the baroreflex sensitivity (BRS).

The limitations of the above studies [32,34] were that the isometric and dynamic RE were not matched for exercise duration and intensity, and force production was not monitored during the two modes of contraction. The BP response, however, is greatly affected by both the intensity and the duration of load [3,35]. In a subsequent study, Chapman et al. [36], using two-leg large extension exercise, equated the intensity (50% MVC), the duration (1 min), and the force generated during one isometric RE protocol and three dynamic RE protocols that varied in the displacement range of the load. In contrast to previous studies [32,34], Chapman et al. [36] observed a smaller increase in heart rate and systolic BP in the isometric than in the dynamic RE protocols, but a greater increase in diastolic BP in isometric compared to dynamic RE protocols. In fact, the magnitude of increase in diastolic BP was greater as the exercise became more static (from 15 cm displacement to 0 cm displacement). Of note, the mean BP (as calculated by us) appears relatively similar at the completion of isometric and dynamic RE (127.5 versus 125 mmHg). The authors also found significant correlations between the increase in heart rate and systolic BP with the distance through which a weight was lifted (r = 0.44 and r = 0.67, respectively). They attributed the greater increase of diastolic BP in the isometric protocol either to the increased mechanical compression of the blood vessels and the subsequent failure of local vasodilation, or to the increased α-adrenergic vasoconstriction within the active muscles and/or in other vascular beds. That study, however, did not examine the possible differences in cardiovascular adjustments associated with increases in mean arterial pressure (MAP) (i.e., contributions of stroke volume, cardiac output, and peripheral resistance) between isometric and dynamic RE.

One of the first studies to compare hemodynamic responses between isometric and isokinetic resistance exercise, another type of dynamic RE, was performed by Haennel et al. [37]. Isometric compared to isokinetic RE produced smaller increases in heart rate and cardiac output; however, similar increases in BP were observed in two protocols. Furthermore, in both protocols, stroke volume did not change compared to baseline levels. Notably, systemic vascular resistance decreased at the end of exercise only in the isokinetic RE protocol, whereas it remained relatively stable in the isometric exercise. Thus, the similar increase in BP during the two exercise modes was mediated by different mechanisms: during isometric exercise, the increase in BP was mainly mediated by the increase in cardiac output (due to increase in heart rate), whereas during isokinetic RE, the BP response was mediated by an increase in cardiac output and a reduction in vascular resistance. The results of this study, however, are greatly limited by the fact that force output was not maintained constant and total workload was not matched between the isometric and isokinetic RE. Ten years later, Iellamo et al. [38] compared the BP and heart rate responses between isokinetic, isotonic, and isometric RE protocols. This was the first study to employ continuous beat-by-beat monitoring of BP and heart rate and to describe both the magnitude as well as the time course of BP changes. Compared to previous studies, the investigators used a larger cohort of participants (10 versus 5–6 in previous studies). The participants performed a submaximal one-leg exercise using isokinetic, isotonic, and isometric RE protocols of equal duration and intensity (1 min exercise, 30 concentric extensions at 40% peak torque for dynamic RE protocols, and 1 min exercise at 40% MVC for isometric RE). The magnitude of increase in BP response, heart rate, and oxygen consumption was not different between the two dynamic RE protocols, however both caused greater responses compared to isometric RE. This is despite the fact that the workload (force × time integral) was two-fold greater in isometric than in dynamic RE protocols. The time-course analysis showed that during both isometric and dynamic RE protocols, there is an initial abrupt increase in pressor and heart rate responses that is followed by a less steep upslope to the end of exercise.

The above studies [33,36,37,38], support the initial view presented by Lewis et al. [32,34]. That is: (i) during RE with small muscle groups, the mode of contraction (static or dynamic) is a minor determinant of cardiorespiratory responses [32,33,34] and (ii) when using large muscle mass, the type of muscle contraction (dynamic versus isometric) certainly affects the magnitude cardiorespiratory responses (i.e., higher heartrate and oxygen consumption in dynamic RE) [34,36,37,38]. The magnitude of the pressor response, however, has been reported as similar in two types of RE [33,34,36,37] (in Chapman et al., only for mean BP), increased in dynamic RE [36,38] (in Chapman et al., only for systolic BP), or increased in isometric RE [36,39] (in Chapman et al., only for diastolic BP). Nevertheless, the conclusions of all the above studies are limited by the fact that the total workload, an important factor to control to accurately compare the magnitude of cardiovascular response between RE protocols, was not matched between isometric and dynamic RE.

In the year 2000, Daniels et al.’s study [29] was the first to emphasize the importance of controlling for workload, in order to adequately compare the cardiovascular responses between isometric and dynamic RE. The study, however, was performed in anesthetized cats using electrical stimulation, thus it was not possible to generalize these finding to humans performing voluntary contraction. Two years later, one of the co-authors of the above study “replicated” the experiment in healthy humans 20 to 51 years of age [40]. The cardiovascular responses were monitored continuously and compared between dynamic (30% of MVC for 180 s and 60% of MVC for 90 s at a rate of 1 repetition/s) and isometric (30% of MVC for 90 s) handgrip exercises equalized for total workload (tension-time integral). In line with previous findings in cats, the authors concluded that when isometric and dynamic RE are matched for active muscle mass (handgrip exercise), intensity (30% of MVC), and total workload, they elicit relatively similar increases in BP, heart rate, and myocardial stress (pulse-rate product). However, increasing the tension in dynamic contraction compared to isometric (60% versus 30% of MVC) elicits a greater cardiovascular response despite similar workloads in the two contraction modes. In line with this, Vedsted et al. [41], comparing two intermittent isometric (1 min at 10% and 20% of MVC) and two dynamic (1 min at 10% και 20% of MVC) RE protocols, also reported that the mean arterial BP was not different between isometric and dynamic RE when performed with relatively small muscle mass (an elbow flexors/extensors) and under equivalent workload. The same study also observed greater electromyographic activity in dynamic RE, greater intramuscular pressure in isometric RE, and no differences in muscle oxygenation between the two contraction modes. Of note, in both studies [40,41], RE protocols used relatively small muscle mass, low force, and did not fatigue the participants. Also, Vedsted et al. [41] used older participants and intermittent (not continuous) isometric/dynamic protocols; that is, 4 s contraction intercepted by 4 s resting intervals. Intermittent protocols with resting intervals between contractions (isometric or dynamic) may significantly alter the blood flow pattern that is observed during continuous contractions and cardiovascular responses. It is conceivable that the intermittent approach would reduce the detrimental effects of increased intramuscular pressure on muscle perfusion to a greater extent during isometric contraction, as the dynamic contraction already alternates muscle contraction with periods of relaxation.

One of the most well-designed studies comparing the BP responses between the two modes of RE using small muscle mass was performed by Edwards et al. [42]. For the first time, the authors compared the effects of static and dynamic muscle contractions on changes in central (aortic) BP. Young, healthy individuals performed handgrip exercise using isometric (90 s at 30% of MVC) and dynamic (1 contraction/s for 180 s at 30% MVC) contractions. The pros of this study were that both RE protocols were performed by the same muscle group, at the same peak tension, and at the same workload. Their main conclusions were that (i) the magnitude of increase in peripheral and central BP, in the augmentation index, and in systolic- and diastolic-pressure time indices were similar at the end of isometric and dynamic contractions and (ii) the peripheral and central BP responses to post-exercise ischemia were similar in the two RE protocols. The above findings suggest that when the same small muscle groups (handgrip exercise), peak tension, and workload are used, the two types of contraction elicit similar increases in peripheral BP, central (aortic) BP, arterial stiffness, work of the heart and coronary perfusion, and metaboreceptor-induced activation of the exercise pressor reflex.

In contrast to the results of the above studies, Koba et al. [43], Arimoto et al. [39], and Yamauchi et al. [44] documented that the magnitude of increase in MAP was greater during isometric than during dynamic RE. All three studies used intermittent/large muscle mass exercise (one or/and two knee-extension exercise). In Koba et al. [43], young, healthy participants performed the isometric and dynamic RE protocols at equivalent workloads using one-leg knee extension. In opposition to previous findings by Stebbins’ et al. [40], Koba et al., reported that the higher BP response in isometric compared to dynamic RE performed at equivalent workload was achieved by exercising either at similar tension (by increasing the exercise duration) or with similar duration (by increasing the exercise intensity). Their results, however, supported previous findings [40] that blood flow and oxygen consumption are greater during dynamic RE. The similarities [40] or the dissimilarities [43] in BP responses between the two contraction modes were attributed to analogous changes in muscle metabolite accumulations and neural activation by muscles’ metaboreceptors or mechanoreceptors. The major difference in the experimental design and possibly the main reason for the opposing results between the two studies for the differences in pressor response between isometric and dynamic RE is the active muscle mass. Stebbins et al. [40] exercised small muscle mass (handgrip exercise), whereas Koba et al. [43] performed the exercise protocols using intermediate muscle mass (one-leg knee extension). In two subsequent studies, Arimoto et al., and Yamauchi et al., compared BP [39,44] and other cardiorespiratory responses [39] during isometric and dynamic RE protocols [39]. Both studies were conducted in healthy, young individuals. The first study [39] employed unilateral and bilateral leg press at two exercise intensities (20% and 40% of MVC) for each type of contraction, and the second study [44] employed isometric and dynamic knee-extensions contractions that differ in tension. Arimoto et al. [39] observed a different pattern of increase in cardiorespiratory parameters between the two types of RE protocols. In dynamic RE, the initial abrupt increase in cardiorespiratory responses was followed by a plateau, whereas during the isometric RE, the cardiorespiratory responses continuously increased until the end of exercise. The magnitude of response in BP (systolic and diastolic) [39,44] and rate-pressure product [39] was greater during isometric compared with dynamic RE. Unfortunately, the findings of these two studies [39,44] are limited by the fact that RE protocols were not matched for total workload and in one study [44], the isometric protocol was performed at considerably higher tension (force).

**Table 1:** Summary of human studies that used small muscle mass (handgrip or arm exercise) to directly compare the hemodynamic responses between isometric and dynamic resistance exercise protocols.

Study	Participants	Muscle Mass	Workload (TTI)	Study Design	Results
Lewis [32]	6 healthy males (26 ± 3 years)	Small	Not Measured	**Handgrip (to Fatigue)** Isometric: 25% MVC Dynamic: 20–40 reps/min 11 kg	↑SBP, ↑DBP, ↑MAP: Isometric = Dynamic ↑HR, ↑CO: Isometric = Dynamic →SV: Isometric = Dynamic ↑TPR only in Isometric ↑VO_2_: Isometric = Dynamic
Lewis [34]	6 healthy males (27 ± 7 years)	Small	Not Measured	**Handgrip (to Fatigue)** Isometric: 25% MVC Dynamic: 33-40 reps/min 11 kg	↑SBP, ↑DBP ↑MAP: Isometric = Dynamic ↑HR, ↑CO: Isometric = Dynamic →SV, →TPR: Isometric = Dynamic
Haennel [37]	5 healthy males (26 ± 3 years)	Small	Not Measured	**Elbow Extension** Isometric: 20 s maximal contraction Isokinetic: 20 s as fast as possible (3 speeds)	↑MAP: Isometric = Isokinetic →SV: Isometric = Isokinetic ↑HR, ↑CO, ↑RPP: Isometric < Isokinetic ↓ SVR: Only in high speed Isokinetic > Isometric
Louhevaara [33]	21 healthy males (33 ± 6 years)	Small	Not Measured	**Handgrip (to Fatigue)** Isometric: 50% MVC Dynamic: 50% MVC (50 reps/min)	↑SBP, ↑DBP: Isometric =Dynamic ↑HR: Isometric = Dynamic ↑VO_2_, ↑VE: Isometric = Dynamic
Vedsted [41]	8 healthy, 1 male and 7 females (45-69 years)	Small	Equivalent in isometric and dynamic protocols	**Elbow Flexion/Extension** Isometric: 1 min, 10% MVC (4 s contraction, 4 s rest) Isometric: 1 min, 20% MVC (4 s contraction, 4 s rest) Dynamic: 1 min, 10% MVC (2 s concentric, 2 s eccentric, 2 s rest) Dynamic: 1 min, 20% MVC (2 s concentric, 2 s eccentric, 2 s rest)	↑SBP: Isometric = Dynamic ↑DBP: Isometric = Dynamic ↑Intramuscular pressure: Isometric > Dynamic ↓ Muscle Oxygenation: Isometric = Dynamic EMG and MMG: Isometric < Dynamic
Stebbins [40]	10 healthy, 7 males and 3 females (20-51 years)	Small	Equivalent among 3 protocols	**Handgrip** Isometric: 90 s, 30% MVC Dynamic: 180 s, 30% MVC, 1 rep/s Dynamic: 90 s, 60% MVC, 1 rep/s	**Similar tension with variable time (equal TTI)** ↑SBP,↑DBP,↑MAP: Isometric = Dynamic ↑HR, ↑RPP: Isometric = Dynamic ↑CO only in Dynamic: Isometric = Dynamic →SV, →SVR: Isometric = Dynamic Blood Flow: Isometric < Dynamic RPE: Isometric = Dynamic **Increased tension in Dynamic with similar time (equal TTI)** ↑SBP,↑DBP,↑MAP: Isometric < Dynamic ↑HR, ↑CO, ↑RPP: Isometric < Dynamic →SV, →SVR: Isometric = Dynamic ↑Blood Flow: Isometric < Dynamic ↑RPE: Isometric < Dynamic
Edwards [42]	14 healthy, 9 males and 5 females (23 ± 19 years)	Small	Equivalent in isometric and dynamic protocols	**Handgrip** Isometric: 90 s, 30% MVC Dynamic: 180 s, 30% MVC, 1 rep/s	↑SBP,↑DBP: Isometric = Dynamic ↑HR: Isometric = Dynamic ↑cSBP,↑cDBP: Isometric = Dynamic ↑Augmentation index: Isometric = Dynamic ↑STI, ↑DTI: Isometric = Dynamic

TTI = Tension-time integral; MVC = Maximal voluntary contraction; SBP = Systolic blood pressure; DBP = Diastolic blood pressure; MAP = Mean arterial pressure; HR = Heartrate; CO = Cardiac output; SV = Stroke volume; TPR = Total peripheral resistance; SVR = Systemic vascular resistance; VO_2_ = Oxygen consumption; RPP = Rate-pulse product; VE = pulmonary ventilation; RPE = Rate of perceived exertion; EMG = Electromyography; MMG = Mechanomyography; STI = Systolic-time index (index of work of the heart); DTI = Diastolic-time index (index of coronary perfusion).

**Table 2:** Summary of human studies that used intermediate/large muscle mass (one-leg and/or two-leg exercise) to directly compare the hemodynamic responses between isometric and dynamic resistance exercise protocols.

Study	Participants	Muscle Mass	Workload (TTI)	Study Design	Results
Lewis [34]	6 healthy males (27 ± 7 years)	Large	Not Measured	**Two-Leg Knee Extension (to Fatigue)** Isometric: 25% MVC, 90° Dynamic: 33-40 reps/min 35 kg	↑SBP,↑MAP: Isometric = Dynamic ↑DBP: Isometric > Dynamic ↑HR: Isometric = Dynamic↑CO: Isometric < Dynamic ↑SV, ↓TPR: Only in Dynamic > Isometric ↑VO_2_: Isometric < Dynamic
Chapman [36]	5 healthy females (21–22 years)	Large	Uncertain	**Two-Leg Knee Extension** Isometric: 1 min, 50% MVC 3 Dynamic: 1 min (50 reps), 50% MVC (Displacement: 5, 10, 15 cm)	↑SBP: Isometric < Dynamic ↑DBP: Isometric > Dynamic ↑MAP*: Isometric = Dynamic ↑HR: Isometric < Dynamic
Haennel [37]	5 healthy males (26 ± 3 years)	Intermediate	Not Measured	**One-Leg Knee Extension** Isometric: 20 s maximal contraction Isokinetic: 20 s as fast as possible (3 speeds)	↑MAP: Isometric = Dynamic ↑HR: Isometric < Dynamic ↑CO: Isometric < Dynamic →SV: Isometric = Dynamic ↓ SVR: Only in Dynamic > Isometric ↑RPP: Isometric = Dynamic
Iellamo [38]	10 healthy males (22–42 years)	Intermediate	Not Equivalent Isometric > Both Dynamic	**One-Leg Knee Extension** Isometric: 1 min, 40% MVC Isokinetic: 30 reps, 40% Peak Torque Isotonic: 30 reps, 40% MVC	↑SBP: Isometric < Isokinetic = Isotonic ↑DBP: Isometric < Isokinetic = Isotonic ↑HR: Isometric < Isokinetic = Isotonic ↑VO_2_, ↑VE: Isometric < Isokinetic = Isotonic
Koba [43]	9 healthy, 4 males and 5 females (27 ± 9 years)	Intermediate	Equivalent among 3 protocols	**One-Leg Knee Extension** Isometric: 2 min sustained, 20% MVC Isometric: 2 min sustained, 40% MVC Dynamic: 4 min, 40% MVC (1 s contraction and 1 s relaxation)	**Similar tension-variable time (equal TTI)** ↑MAP: Isometric > Dynamic ↑HR: Isometric = Dynamic ↑Blood Flow ↑VO_2_: Isometric < Dynamic **Increased tension-similar time (equal TTI)** ↑MAP: Isometric > Dynamic ↑HR: Isometric = Dynamic ↑Blood Flow ↑VO_2_: Isometric < Dynamic
Arimoto [39]	7 healthy males (20 ± 1 years)	Intermediate Large	Not Measured	**One- and Two-Leg Knee Extension** Isometric: 6 min, 20% MVC, angle 90º Isometric: 3 min, 40% MVC, angle 90º Dynamic: 6 min, 20% MVC, range 90º Dynamic: 6 min, 40% MVC, range 90º	**One-Leg Knee Extension (20 and 40% MVC)** ↑SBP, ↑DBP, ↑RPP: Isometric > Dynamic ↑HR: Isometric ≥ Dynamic ↑VO_2_: Isometric < Dynamic **Two-Leg Knee Extension (20 and 40% MVC)** ↑SBP, ↑RPP: Isometric = Dynamic (at 20%) ↑SBP, ↑RPP: Isometric > Dynamic (at 40%) ↑DBP: Isometric > Dynamic ↑HR: Isometric ≥ Dynamic ↑VO_2_: Isometric < Dynamic
Yamauchi [44]	18 healthy participants (19 ± 1 years)	Intermediate	Not Measured	**One-Leg Knee Extension** Isometric: 1 contraction, 100% F_0_ Dynamic: 1 contraction, 12% F_0_ Dynamic: 1 contraction, 22% F_0_ Dynamic: 1 contraction, 33% F_0_ Dynamic: 1 contraction, 46% F_0_ Dynamic: 1 contraction, 66% F_0_	↑MAP: Isometric > all Dynamic

TTI = Tension-time integral; MVC = Maximal voluntary contraction; F_0_ = maximal isometric force; SBP = Systolic blood pressure; DBP = Diastolic blood pressure; MAP = Mean arterial pressure; MAP* = Mean arterial pressure as calculated by us; HR = Heartrate; CO = Cardiac output; SV = Stroke volume; TPR = Total peripheral resistance; SVR = Systemic vascular resistance; VO_2_ = Oxygen consumption; RPP = Rate-pulse product.

## 4. Discussion

This review presented the studies that directly compared the BP response and cardiovascular responses between the two types of RE. Overall, the results of these studies suggest that in healthy individuals, (i) isometric and dynamic RE protocols using the same small muscle mass (i.e., handgrip and elbow flexion/extension) evoke similar increases in BP (irrespective of contraction mode), (ii) there are controversial findings regarding the magnitude of BP increase between isometric and dynamic RE protocols when the same intermediate and/or large muscle mass was used, and (iii) the cardiovascular adjustments involved in the BP increase (i.e., cardiac output, peripheral resistance) appear different in isometric compared to dynamic RE.

We hasten to add that only a few human studies directly compared the effects of isometric and dynamic RE on BP (12 studies) using RE protocols of similar muscle mass. Importantly, only four of these equalized the two types of RE for both total workload and muscle mass [40,41,42,43], a vital experimental approach to accurately compare the cardiovascular response between the two types of RE. Five studies, which compared the isometric and dynamic RE protocols, included other cardiovascular measures as well [32,34,37,40,42]. Seven studies in humans employed RE protocols using the small muscle mass [32,33,34,37,40,41,42] and six used large muscle mass [36,37,38,39,43,44] (Table 1 and Table 2). Finally, all studies that compared the cardiovascular responses between isometric and dynamic RE employed healthy individuals and not a typical RE protocol (multiple sets). This precludes the comparison of cardiovascular risk associated with the two modes of RE for individuals with chronic diseases, particularly when performing RE protocols based on recent recommendations for multiple sets. This is of major importance considering a different pattern of cardiovascular changes between isometric and dynamic RE protocols [38,39].

Studies that used RE protocols with small muscle mass showed that both isometric and dynamic RE were associated with elevations in systolic BP (about 25–40 mmHg) and diastolic BP (about 15–30 mmHg). This magnitude of increase in systolic and diastolic BP during RE protocols appears similar and greater respectively, compared to those observed during moderate intensity aerobic exercise using large muscle mass. However, when isometric and dynamic RE protocols using small muscle mass (i.e., handgrip and elbow flexion/extension) were compared, equal increases in BP were found. Thus, the type of muscle contraction (dynamic versus isometric) is not the major determinant of the magnitude of pressor response when using small muscle mass RE. This is well supported by all studies that directly compared the two types of muscle contraction [29,32,33,34,37,40,41,42]. This view is strengthened by the fact that three of the above studies employed a study design that controlled for both the intensity and total workload of RE protocols [40,41,42]. It should be noted, however, that in two of these studies, the duration of contraction was not matched between the two types of RE [40,42] and in the other study, intermittent isometric exercise allowed a 4 second relaxation between contractions [41]. Increasing the duration of RE may additionally tax the cardiovascular system [3,35], while isometric intermittent protocols with resting intervals between contractions may allow a great reperfusion of the muscle, which may reduce the detrimental effects of increased intramuscular pressure on muscle blood flow during isometric RE.

Active muscle mass is an important contributor to cardiovascular responses during RE. The increase in cardiovascular response to RE is associated with the muscle mass involved in the contraction [34,37,45]. In contrast to the consistent results of studies using small muscle mass, the studies that compared the magnitude of BP responses between isometric and dynamic RE protocols using the same large muscle mass produced equivocal results [36,37,38,39,43,44]. That is, two studies showed no difference in MAP response [36,37] between isometric and dynamic RE, one study showed that isokinetic and isotonic dynamic one-leg knee extension elicit greater increase in BP response compared to isometric contraction [38], while others documented that isometric compared to dynamic leg press causes a greater rise in mean BP [43,44] and in both systolic and diastolic BP [39]. Earlier work showed that systolic BP increases to a greater extent in two-leg dynamic RE compared to static, while diastolic BP progressively increases as the speed of contraction decreases (the contraction becomes more static) [36]. The limitations of studies using large muscle mass are that all studies (except Koba et al.) did not appear to match the two RE protocols for total workload and one study used significantly greater tension in isometric compared to dynamic contractions [44]. Both factors are crucial for the magnitude of the BP response. Based on the above, the differences between isometric and dynamic RE protocols on the BP and other cardiovascular responses during large muscle mass exercise are not clear and more well-controlled investigations are needed. In general, there is a consensus among studies that the magnitude of increase in heart rate and oxygen consumption are lower during isometric RE compared with dynamic RE performed with similar muscle mass and intensity. This is even more evident when the RE protocols are performed with larger muscle mass.

### 4.1. Mechanisms Controlling the Increase in BP during Isometric and Dynamic Resistance Exercise

**Figure 2:**
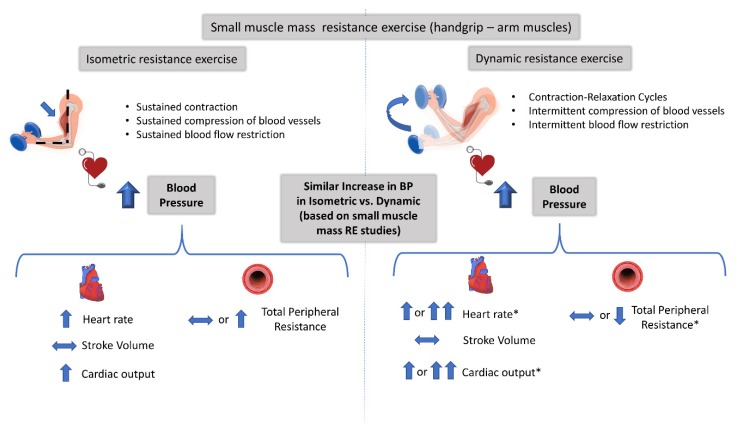
Blood pressure (BP) response and cardiovascular adjustments controlling the BP response, during small muscle mass isometric and dynamic resistance exercise (RE). There is a consensus among studies that the magnitude of BP response during small muscle mass isometric exercise is similar to that in dynamic RE. Arrows denote the direction of the response during each mode of exercise. Double arrows denote a greater response in dynamic RE versus isometric. *some studies report similar and other studies increased or different response in dynamic RE versus isometric exercise.

**Figure 3:**
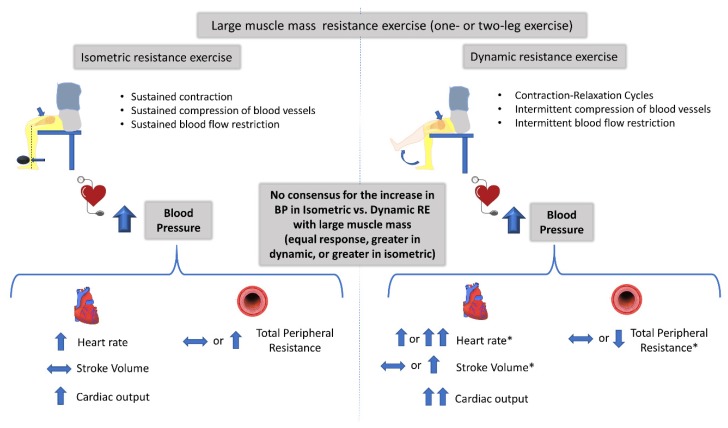
Blood pressure (BP) response and cardiovascular adjustments controlling the BP response, during intermediate or large muscle mass isometric and dynamic resistance exercise (RE) with similar load. Studies using intermediate- or large-muscle mass (one- or two-leg muscles) report inconsistent results for the magnitude of the BP response between the two types of RE. Three studies report an equal BP response, two studies report a greater BP increase in isometric, and one study reports a greater BP increase in dynamic RE. Arrows denote the direction of the response during each mode of exercise. Double arrows denote greater response in dynamic RE versus isometric. *some studies report similar and other studies increased or different response in dynamic RE versus isometric exercise at similar tension.

Three scientific groups have directly compared the cardiovascular responses between isometric and dynamic RE and provided a nice set of data for the principal components that determine the pressor response [32,34,37,40]. From these studies, only in Stebbins et al. [40] were the RE protocol matched for workload. It appears that the increase in BP during isometric and dynamic RE protocols with small muscle mass are due to increases in cardiac output (as a result of greater increases in heartrate, as stroke volume remained virtually the same and vascular resistance did not change during the two contraction modes (at 30% MVC). The authors attributed the unexpected lack of changes in stroke volume and in peripheral resistance during the dynamic RE to the small active muscle mass (handgrip exercise). The fact that this study included participants of both genders and of different ages might have also affected the findings regarding the determinants of BP response to RE, as shown in previous studies [46,47,48]. The same conclusion had been reached earlier by Lewis et al. [32,34], even though the investigators did not control the workload between the two RE protocols. They clearly showed that the exercising muscle mass affects the circulatory adjustments (i.e., stroke volume, cardiac output, and peripheral resistance) that contribute to the pressor response, especially during dynamic RE. For example, the increase in stroke volume and the reduction in peripheral resistance were observed only during dynamic and large muscle mass RE. This is in line with previous studies reporting that RE with small muscle mass (compared to large muscle mass RE) results in attenuated stroke volume and peripheral resistance responses despite the large BP response [34,49]. Another mechanism that may contribute to the lack of significant changes in stroke volume during isometric RE, irrespective of exercising muscle mass [34], may be related to a reduction in cardiac preload and to an increase in afterload [3]. This is possibly a result of the reduced muscle pump and venous return due to greater intramuscular pressure [41] partially occluding muscle vasculature and of the higher intrathoracic pressure [50] occurring during isometric RE. In support of this, muscle blood flow was significantly lower during isometric compared to dynamic RE with small and large exercising muscle mass [40,43]. The described mechanisms involved in the increase in BP during isometric and dynamic RE protocols using small muscle mass and intermediate/large muscle mass are depicted in Figure 2 and Figure 3, respectively.

Three neural mechanisms have been proposed to explain either the differences or the similarities for the increase in the pressor response and cardiovascular adjustments between isometric and dynamic RE: (i) the stimulation of neural reflexes arising from the exercising muscle named mechanoreflex and metaboreflex (as a result of mechanical movement/compression and increased metabolites within the muscle, respectively) [51,52,53], (ii) the “central command” (activation of centrally mediated efferent pathways) originating from the brain [52,54], and (iii) the resetting of baroreflex, which is influenced by both the “central command” and the muscle reflexes (chemoreflex and mechanoreflex) [55,56,57,58]. To the best of our knowledge, only three studies compared the metaboreflex [40,42,43] and one the “central command” [40] between isometric and dynamic RE. Two studies using handgrip exercise showed that the equal rise in BP during isometric and dynamic RE, matched for intensity and workload, was accompanied by similar activation of the central command (estimated by the rate of perceived exertion, RPE) [40] and muscle metaboreflex in the two types of RE [40,42]. The conclusion regarding the contribution of “central command” to BP response should be interpreted with caution due to (i) the methodology used (RPE) and (ii) the fact that the central command plays a role in regulating muscle sympathetic activity only during intense (>50 of MVC) and intermittent (not sustained) isometric exercise [54,59]. In contrast, the other study using one-leg exercise concluded that the greater pressor response in isometric compared to dynamic RE protocol must be partially attributed to greater metaboreflex activation (increased MAP in post-exercise occlusion) in isometric and unlikely to differences in central command between the two modes of RE [43].

Catecholamines are also involved in cardiovascular adjustments during exercise [60]. The catecholamine release, however, during isometric and dynamic exercise is largely controlled by the activation of both central nervous system and muscle neural reflexes (metaboreflex and mechanoreflex) [61,62,63,64]. This is the reason that circulating catecholamines are used as an index of sympathetic activation [63,65]. Apart from the effect of intensity and duration of exercise [63], the magnitude of catecholamine and endocrine responses are proportional to active muscle mass [49,62,64]. Lewis et al., compared adrenergic response after isometric and dynamic RE performed with large (two-knee extension) and small (handgrip) muscle mass [32,34]. They observed that (i) circulating norepinephrine (NE) increased after both static and dynamic RE using small or large muscle mass [32,34], (ii) the magnitude of increase in NE was greater in dynamic RE than in static, irrespective of active muscle mass [34], (iii) the increase was more pronounced during large (two-knee extension) compared to small (handgrip) muscle mass RE [34], and (iv) during dynamic RE, the increase in plasma NE was two-fold higher in large compared to small muscle mass RE [34]. On the other hand, after ten weeks of resistance training, the resting plasma NE concentration increased more after upper limb training than after lower limb training [66].

### 4.2. Safety of Isometric versus Dynamic Resistance Exercise

Although dynamic RE has been widely recommended by medical associations as an adjunct to endurance training for the prevention and treatment of chronic diseases, the health community has been reluctant to promote isometric RE. In fact, only recently, the American Heart Association was the first to include isometric exercise in guidelines as a complementary prevention and therapeutic strategy [20]. This hesitation is mainly based on the notion that isometric RE is associated with greater elevations in BP compared to dynamic exercise, increasing the risk for possible cardiovascular and cerebrovascular complications, especially in middle-aged, elderly, and hypertensive individuals [28]. The view, however, that isometric produces greater pressor response compared to dynamic exercise is mostly a result of studies comparing isometric with moderate intensity dynamic aerobic (endurance) exercise.

As presented in this review, most studies have shown that isometric RE produces equal BP responses to dynamic RE when performed with similar muscle mass and equivalent workloads. These studies, however, were conducted in healthy individuals and the findings should not be inferred to other populations. Thus, in terms of afterload, both RE protocols appear to produce similar stress to the heart, although possibly higher compared to moderate intensity aerobic exercise. However, in stage I hypertensives, systolic BP values reported during a fatiguing isometric RE (4 sets of 2 min wall squats) did not exceed the current American College of Sports Medicine guidelines for aerobic exercise termination, while the diastolic BP briefly exceeded 115 mmHg [67]. In general, heartrate, pressure-rate product (an index of myocardial work), as well as cardiac output, increase more in dynamic RE than in isometric RE (Table 1 and Table 2). The higher diastolic BP and the lower pressure-rate product during isometric RE is consistent with a more favorable myocardial oxygen supply to demand balance [35,68,69] and a lower incidence of ischemia [69,70,71]. Indeed, isometric handgrip caused less cardiovascular symptoms (ST changes, arrhythmias) compared to a maximal treadmill test in patients with heart disease [68,70,71,72,73,74]. However, patients with poor left ventricular function may develop wall-motion abnormalities during isometric or combined isometric and dynamic exercise. As of now, there is no evidence from the few published studies that the transient increase in BP during isometric exercise is associated with a higher risk for acute cardiovascular events [20,71,75], at least in patients with normal left ventricular function. In any case, RE and especially static exercise should be performed with specific instructions to avoid the Valsalva maneuver, since this maneuver results in significant increases in systolic blood pressure due to increased intrathoracic pressure [76]. Future trials are needed to evaluate the cardiovascular risks associated with the transient elevation in BP during isometric RE in different populations.

## 5. Conclusions

Only a few human studies (twelve in total) have directly compared the BP response between isometric and dynamic RE, and even less equalized the two RE protocols for total workload and muscle mass, both important factors to accurately compare the cardiovascular response between the two modes of RE. Notably, all studies that compared the cardiovascular responses between isometric and dynamic RE employed healthy individuals and did not use a typical RE protocol (multiple sets). Studies using RE protocols with small muscle mass (i.e., handgrip and arm) agree that the magnitude of BP response is not different between isometric and dynamic RE. In contrast, studies using large muscle mass (leg muscles) failed to produce a consensus for the magnitude of BP response between the two modes of RE, reporting equivocal results. Thus, when the same muscles and workloads are used when comparing isometric and dynamic RE, the magnitude of BP response is more comparable to what is commonly believed. Different cardiovascular adjustments contribute to this relatively similar BP response during dynamic and isometric RE. The magnitude of increase in cardiovascular response to RE depends on the active muscle mass involved in the contraction, irrespective of the mode of contraction (isometric or dynamic). Finally, isometric RE challenges to a lower extent the oxidative metabolism compared to dynamic RE. To the best of our knowledge, no study has directly compared cardiac symptoms, wall motion disturbances, signs of ischemia, and/or the incidence of arrhythmias between the two modes of RE under equivalent workload conditions. Clearly, more data are needed to establish the safety of isometric versus dynamic RE in healthy individuals and in individuals with chronic disease, particularly using recommended RE protocols consisting of multiple sets.

## References

[1] Braith R.W., Beck D.T. (2008). Resistance exercise: Training adaptations and developing a safe exercise prescription. Heart Fail. Rev..

[2] Garber C.E., Blissmer B., Deschenes M.R., Franklin B.A., Lamonte M.J., Lee I.M., Nieman D.C., Swain D.P. (2011). American College of Sports Medicine position stand. Quantity and quality of exercise for developing and maintaining cardiorespiratory, musculoskeletal, and neuromotor fitness in apparently healthy adults: Guidance for prescribing exercise. Med. Sci. Sports Exerc..

[3] Williams M.A., Haskell W.L., Ades P.A., Amsterdam E.A., Bittner V., Franklin B.A., Gulanick M., Laing S.T., Stewart K.J. (2007). Resistance exercise in individuals with and without cardiovascular disease: 2007 update: A scientific statement from the American Heart Association Council on Clinical Cardiology and Council on Nutrition, Physical Activity, and Metabolism. Circulation.

[4] Cornelissen V.A., Fagard R.H., Coeckelberghs E., Vanhees L. (2011). Impact of resistance training on blood pressure and other cardiovascular risk factors: A meta-analysis of randomized, controlled trials. Hypertension.

[5] Chrysant S.G. (2010). Current evidence on the hemodynamic and blood pressure effects of isometric exercise in normotensive and hypertensive persons. J. Clin. Hypertens..

[6] Pescatello L.S., Franklin B.A., Fagard R., Farquhar W.B., Kelley G.A., Ray C.A. (2004). American College of Sports Medicine position stand. Exercise and hypertension. Med. Sci. Sports Exerc..

[7] Topp R., Woolley S., Hornyak J., Khuder S., Kahaleh B. (2002). The effect of dynamic versus isometric resistance training on pain and functioning among adults with osteoarthritis of the knee. Arch. Phys. Med. Rehabil..

[8] Pluim B.M., Zwinderman A.H., van der Laarse A., van der Wall E.E. (2000). The athlete’s heart. A meta-analysis of cardiac structure and function. Circulation.

[9] Fagard R.H. (1996). Athlete’s heart: A meta-analysis of the echocardiographic experience. Int. J. Sports Med..

[10] Umpierre D., Stein R. (2007). Hemodynamic and vascular effects of resistance training: Implications for cardiovascular disease. Arq. Bras. Cardiol..

[11] Tanimoto M., Ishii N. (2006). Effects of low-intensity resistance exercise with slow movement and tonic force generation on muscular function in young men. J. Appl. Physiol..

[12] Mitchell J.H., Haskell W., Snell P., Van Camp S.P. (2005). Task Force 8: Classification of sports. J. Am. Coll. Cardiol..

[13] Sadamoto T., Bonde-Petersen F., Suzuki Y. (1983). Skeletal muscle tension, flow, pressure, and EMG during sustained isometric contractions in humans. Eur. J. Appl. Physiol. Occup. Physiol..

[14] Kagaya A., Ogita F. (1992). Blood flow during muscle contraction and relaxation in rhythmic exercise at different intensities. Ann. Physiol. Anthropol..

[15] Radegran G. (1997). Ultrasound Doppler estimates of femoral artery blood flow during dynamic knee extensor exercise in humans. J. Appl. Physiol..

[16] MacDougall J.D., Tuxen D., Sale D.G., Moroz J.R., Sutton J.R. (1985). Arterial blood pressure response to heavy resistance exercise. J. Appl. Physiol..

[17] Wernbom M., Augustsson J., Thomee R. (2007). The influence of frequency, intensity, volume and mode of strength training on whole muscle cross-sectional area in humans. Sports Med..

[18] Millar P.J., McGowan C.L., Cornelissen V.A., Araujo C.G., Swaine I.L. (2014). Evidence for the role of isometric exercise training in reducing blood pressure: Potential mechanisms and future directions. Sports Med..

[19] Carlson D.J., Dieberg G., Hess N.C., Millar P.J., Smart N.A. (2014). Isometric exercise training for blood pressure management: A systematic review and meta-analysis. Mayo Clin. Proc..

[20] Brook R.D., Appel L.J., Rubenfire M., Ogedegbe G., Bisognano J.D., Elliott W.J., Fuchs F.D., Hughes J.W., Lackland D.T., Staffileno B.A. (2013). Beyond medications and diet: Alternative approaches to lowering blood pressure: A scientific statement from the american heart association. Hypertension.

[21] Whelton P.K., Carey R.M., Aronow W.S., Casey D.E., Collins K.J., Dennison Himmelfarb C., DePalma S.M., Gidding S., Jamerson K.A., Jones D.W. (2018). 2017 ACC/AHA/AAPA/ABC/ACPM/AGS/APhA/ASH/ASPC/NMA/PCNA Guideline for the Prevention, Detection, Evaluation, and Management of High Blood Pressure in Adults: Executive Summary: A Report of the American College of Cardiology/American Heart Association Task Force on Clinical Practice Guidelines. Circulation.

[22] Sharman J.E., Smart N.A., Coombes J.S., Stowasser M. (2019). Exercise and sport science australia position stand update on exercise and hypertension. J. Hum. Hypertens..

[23] Compton D., Hill P.M., Sinclair J.D. (1973). Weight-lifters’ blackout. Lancet.

[24] Goetting M.G., Swanson S.E. (1987). Massive hemorrhage into intracranial neurinomas. Surg. Neurol..

[25] Haykowsky M.J., Findlay J.M., Ignaszewski A.P. (1996). Aneurysmal subarachnoid hemorrhage associated with weight training: Three case reports. Clin. J. Sport Med..

[26] Pott F., Van Lieshout J.J., Ide K., Madsen P., Secher N.H. (2003). Middle cerebral artery blood velocity during intense static exercise is dominated by a Valsalva maneuver. J. Appl. Physiol..

[27] Vlak M.H., Rinkel G.J., Greebe P., van der Bom J.G., Algra A. (2012). Trigger factors for rupture of intracranial aneurysms in relation to patient and aneurysm characteristics. J. Neurol..

[28] Matsuda M., Watanabe K., Saito A., Matsumura K., Ichikawa M. (2007). Circumstances, activities, and events precipitating aneurysmal subarachnoid hemorrhage. J. Stroke Cerebrovasc. Dis..

[29] Daniels J.W., Stebbins C.L., Longhurst J.C. (2000). Hemodynamic responses to static and dynamic muscle contractions at equivalent workloads. Am. J. Physiol. Regul. Integr. Comp. Physiol..

[30] Greer M., Dimick S., Burns S. (1984). Heartrate and blood pressure response to several methods of strength training. Phys. Ther..

[31] Kordi R., Mazaheri R., Rostami M., Mansournia M.A. (2012). Hemodynamic changes after static and dynamic exercises and treadmill stress test; different patterns in patients with primary benign exertional headache?. Acta Med. Iran..

[32] Lewis S.F., Taylor W.F., Bastian B.C., Graham R.M., Pettinger W.A., Blomqvist C.G. (1983). Haemodynamic responses to static and dynamic handgrip before and after autonomic blockade. Clin. Sci..

[33] Louhevaara V., Smolander J., Aminoff T., Korhonen O., Shen N. (2000). Cardiorespiratory responses to fatiguing dynamic and isometric hand-grip exercise. Eur. J. Appl. Physiol..

[34] Lewis S.F., Snell P.G., Taylor W.F., Hamra M., Graham R.M., Pettinger W.A., Blomqvist C.G. (1985). Role of muscle mass and mode of contraction in circulatory responses to exercise. J. Appl. Physiol..

[35] Bjarnason-Wehrens B., Mayer-Berger W., Meister E.R., Baum K., Hambrecht R., Gielen S. (2004). Recommendations for resistance exercise in cardiac rehabilitation. Recommendations of the German Federation for Cardiovascular Prevention and Rehabilitation. Eur. J. Cardiovasc. Prev. Rehabil..

[36] Chapman J.H., Elliott P.W. (1988). Cardiovascular effects of static and dynamic exercise. Eur. J. Appl. Physiol. Occup. Physiol..

[37] Haennel R.G., Snydmiller G.D., Teo K.K., Greenwood P.V., Quinney H.A., Kappagoda C.T. (1992). Changes in blood pressure and cardiac output during maximal isokinetic exercise. Arch. Phys. Med. Rehabil..

[38] Iellamo F., Legramante J.M., Raimondi G., Castrucci F., Damiani C., Foti C., Peruzzi G., Caruso I. (1997). Effects of isokinetic, isotonic and isometric submaximal exercise on heartrate and blood pressure. Eur. J. Appl. Physiol. Occup. Physiol..

[39] Arimoto M., Kijima A., Muramatsu S. (2005). Cardiorespiratory response to dynamic and static leg press exercise in humans. J. Physiol. Anthropol. Appl. Hum. Sci..

[40] Stebbins C.L., Walser B., Jafarzadeh M. (2002). Cardiovascular responses to static and dynamic contraction during comparable workloads in humans. Am. J. Physiol. Regul. Integr. Comp. Physiol..

[41] Vedsted P., Blangsted A.K., Sogaard K., Orizio C., Sjogaard G. (2006). Muscle tissue oxygenation, pressure, electrical, and mechanical responses during dynamic and static voluntary contractions. Eur. J. Appl. Physiol..

[42] Edwards D.G., Mastin C.R., Kenefick R.W. (2008). Wave reflection and central aortic pressure are increased in response to static and dynamic muscle contraction at comparable workloads. J. Appl. Physiol..

[43] Koba S., Hayashi N., Miura A., Endo M., Fukuba Y., Yoshida T. (2004). Pressor response to static and dynamic knee extensions at equivalent workload in humans. Jpn. J. Physiol..

[44] Yamauchi J., Nakayama S., Ishii N. (2008). Blood pressure response to force-velocity properties of the knee-hip extension movement. Eur. J. Appl. Physiol..

[45] Mitchell J.H., Payne F.C., Saltin B., Schibye B. (1980). The role of muscle mass in the cardiovascular response to static contractions. J. Physiol..

[46] Hart E.C., Charkoudian N., Wallin B.G., Curry T.B., Eisenach J.H., Joyner M.J. (2009). Sex differences in sympathetic neural-hemodynamic balance: Implications for human blood pressure regulation. Hypertension.

[47] Lovell D.I., Cuneo R., Gass G.C. (2011). The blood pressure response of older men to maximum and sub-maximum strength testing. J. Sci. Med. Sport.

[48] Joyner M.J., Wallin B.G., Charkoudian N. (2016). Sex differences and blood pressure regulation in humans. Exp. Physiol..

[49] Blomqvist C.G., Lewis S.F., Taylor W.F., Graham R.M. (1981). Similarity of the hemodynamic responses to static and dynamic exercise of small muscle groups. Circ. Res..

[50] MacDougall J.D., McKelvie R.S., Moroz D.E., Sale D.G., McCartney N., Buick F. (1992). Factors affecting blood pressure during heavy weight lifting and static contractions. J. Appl. Physiol..

[51] Boushel R. (2010). Muscle metaboreflex control of the circulation during exercise. Acta Physiol..

[52] Mitchell J.H., Kaufman M.P., Iwamoto G.A. (1983). The exercise pressor reflex: Its cardiovascular effects, afferent mechanisms, and central pathways. Annu. Rev. Physiol..

[53] Joyner M.J., Nauss L.A., Warner M.A., Warner D.O. (1992). Sympathetic modulation of blood flow and O_2_ uptake in rhythmically contracting human forearm muscles. Am. J. Physiol..

[54] Victor R.G., Secher N.H., Lyson T., Mitchell J.H. (1995). Central command increases muscle sympathetic nerve activity during intense intermittent isometric exercise in humans. Circ. Res..

[55] Gallagher K.M., Fadel P.J., Smith S.A., Stromstad M., Ide K., Secher N.H., Raven P.B. (2006). The interaction of central command and the exercise pressor reflex in mediating baroreflex resetting during exercise in humans. Exp. Physiol..

[56] Hartwich D., Dear W.E., Waterfall J.L., Fisher J.P. (2011). Effect of muscle metaboreflex activation on spontaneous cardiac baroreflex sensitivity during exercise in humans. J. Physiol..

[57] Hureau T.J., Weavil J.C., Thurston T.S., Broxterman R.M., Nelson A.D., Bledsoe A.D., Jessop J.E., Richardson R.S., Wray D.W., Amann M. (2018). Identifying the role of group III/IV muscle afferents in the carotid baroreflex control of mean arterial pressure and heartrate during exercise. J. Physiol..

[58] Iellamo F., Legramante J.M., Raimondi G., Peruzzi G. (1997). Baroreflex control of sinus node during dynamic exercise in humans: Effects of central command and muscle reflexes. Am. J. Physiol..

[59] Victor R.G., Pryor S.L., Secher N.H., Mitchell J.H. (1989). Effects of partial neuromuscular blockade on sympathetic nerve responses to static exercise in humans. Circ. Res..

[60] Zouhal H., Jacob C., Delamarche P., Gratas-Delamarche A. (2008). Catecholamines and the effects of exercise, training and gender. Sports Med..

[61] Mitchell J.H. (1985). Cardiovascular control during exercise: Central and reflex neural mechanisms. Am. J. Cardiol..

[62] Kjaer M., Secher N.H. (1992). Neural influence on cardiovascular and endocrine responses to static exercise in humans. Sports Med..

[63] Mazzeo R.S. (1991). Catecholamine responses to acute and chronic exercise. Med. Sci. Sports Exerc..

[64] Kjaer M., Secher N.H., Galbo H. (1987). Physical stress and catecholamine release. Baillieres Clin. Endocrinol. Metab..

[65] Seals D.R., Victor R.G. (1991). Regulation of muscle sympathetic nerve activity during exercise in humans. Exerc. Sport Sci. Rev..

[66] Okamoto T., Masuhara M., Ikuta K. (2009). Upper but not lower limb resistance training increases arterial stiffness in humans. Eur. J. Appl. Physiol..

[67] Wiles J.D., Taylor K., Coleman D., Sharma R., O’Driscoll J.M. (2018). The safety of isometric exercise: Rethinking the exercise prescription paradigm for those with stage 1 hypertension. Medicine.

[68] McCartney N. (1999). Acute responses to resistance training and safety. Med. Sci. Sports Exerc..

[69] Bertagnoli K., Hanson P., Ward A. (1990). Attenuation of exercise-induced ST depression during combined isometric and dynamic exercise in coronary artery disease. Am. J. Cardiol..

[70] DeBusk R.F., Valdez R., Houston N., Haskell W. (1978). Cardiovascular responses to dynamic and static effort soon after myocardial infarction. Application to occupational work assessment. Circulation.

[71] Franklin B.A., Bonzheim K., Gordon S., Timmis G.C. (1991). Resistance training in cardiac rehabilitation. J. Cardiopulm. Rehabil..

[72] Chaney R.H., Arndt S. (1983). Comparison of cardiovascular risk in maximal isometric and dynamic exercise. South. Med. J..

[73] Haissly J.C., Messin R., Degre S., Vandermoten P., Demaret B., Denolin H. (1974). Comparative response to isometric (static) and dynamic exercise tests in coronary disease. Am. J. Cardiol..

[74] Kerber R.E., Miller R.A., Najjar S.M. (1975). Myocardial ischemic effects of isometric, dynamic and combined exercise in coronary artery disease. Chest.

[75] Kelley G.A., Kelley K.S. (2010). Isometric handgrip exercise and resting blood pressure: A meta-analysis of randomized controlled trials. J. Hypertens..

[76] Zebrowska A., Gasior Z., Jastrzebski D. (2013). Cardiovascular effects of the valsalva maneuver during static arm exercise in elite power lifting athletes. Adv. Exp. Med. Biol..

